# Micro-Hotplate for Thermocatalytic Gas Sensor Fabricated by Ceramic Laser Micromachining

**DOI:** 10.3390/mi17010059

**Published:** 2025-12-31

**Authors:** Nikolay Samotaev, Gennady Zebrev, Konstantin Oblov, Maya Etrekova, Pavel Dzhumaev, Ivan Obraztsov, Boris Podlepetsky

**Affiliations:** Micro- and Nanoelectronics Department, National Research Nuclear University MEPhI (Moscow Engineering Physics Institute), 115409 Moscow, Russia; gizebrev@mephi.ru (G.Z.); kyoblov@mephi.ru (K.O.); moetrekova@mephi.ru (M.E.); psdzhumaev@mephi.ru (P.D.); yuganinn@yandex.ru (I.O.); bipod45@gmail.com (B.P.)

**Keywords:** thermocatalytic sensor, LEL, ceramics, laser micromachining

## Abstract

Thermocatalytic sensors are used as universal explosion meters for measurement of the Lower Explosive Limit (LEL) of hydrocarbon gases mixtures. Historically, thermo-catalytic sensors, with their bulky “pellistor” design, have been poorly suited for mass production using group microelectronic processing. Another significant challenge for developers of new sensor designs is to minimize power dissipation while enhancing the service life and resistance of catalytic elements to poisoning from silicon–organic and sulfur-containing gases. To meet the specified requirements, we developed a low-power thermocatalytic sensor utilizing ceramic technology, capable of holding the temperature of technology operations up to 900 °C.

## 1. Introduction

Thermocatalytic sensors have established themselves as reliable universal exposure meters for indicating the Lower Explosive Limit (LEL) of hydrocarbon gas mixtures in the air. This capability arises from the direct proportionality between the heat generated by combustion on an active catalytic element at temperatures of 450–500 °C and the total LEL present in the air [1]. Due to this physical–chemical effect, there is currently no direct alternative to thermocatalytic sensors, even though semiconductor, optical, and thermoconductometric gas sensors can measure the concentration of individual gases [2]. Thermocatalytic sensors are mass-produced using two primary technologies: platinum micro-spiral [3,4] and silicon membrane MEMS technology (membrane construction) [5,6]. Notably, many companies that produce thermocatalytic sensors on a large scale often support both technologies, as each has its own advantages and drawbacks [3,4,5,6].

The platinum micro-spiral design, also known as the bead type, offers the advantage of a higher volume of catalytic material due to its volumetric spherical shape. However, despite the use of automation, its production is not carried out in a microelectronic group process; each micro-spiral is manufactured sequentially on the same winding devices. Mass-produced planar sensors utilizing silicon MEMS membranes offer advantages such as reduced energy consumption and faster manufacturing speeds, thanks to the integration of group microelectronics processes on wafers. However, this approach comes with the drawback of a limited volume of catalytic material being deposited on the membrane micro-hotplate, which is located to one side of the membrane due to the specific design choices employed.

In real conditions, the amount of catalytic material determines the sensor’s resistance to poisoning by various silicon–organic and sulfur-containing gaseous substances, that is, its active life [7]. As a rule, chemical poisoning starts from the surface into the catalytic material’s volume, and here the design in the form of a bead with a diameter of hundreds of micrometers [8,9] has the advantage of poisoning the outer layers (surface), leaving the inner layers serviceable. Planar MEMS sensors have a catalytic layer of several dozen micrometers, in which the diffusion of catalytic poisons is carried out immediately to the entire depth. To take into account these factors, companies which produce sensors try to use sorption filters built into the sensor housing as a barrier to the penetration of catalytic poisons, for example, as in the silicon MEMS sensor design [6]. In defense of the currently used technologies, it can be said that there are palliative solutions to maintain the signal stability of the already poisoned sensor [10], for example, recalibration of sensors that have reduced sensitivity due to diffusion limitations arising from the combustion of gases in the internal volume of the package [9], but this approach does not solve the fundamental design problem—a small volume of catalytic material deposited on a micro-hotplate.

Several scientific groups are working to develop a hybrid design that combines the advantages of three-dimensional structures with microelectronic technologies. A common solution involves utilizing a cantilever structure where catalytic material is deposited on both sides of a micro-hotplate. Typically, these thermocatalytic sensor cantilevers are fabricated using either silicon [11] or ceramic technologies [12]. Silicon technology generally allows for smaller cantilever micro-hotplates compared to ceramic alternatives. As the size of the micro-hotplate decreases, notable differences emerge in the manufacturing processes and the methods for depositing the catalytic material.

First of all, this is expressed as a decrease in catalytic material carrier particle size (typically using Al_2_O_3_ [13] or ZrO_2_ [6]); the particle sizes drop from a few micrometers (used for the bead-type design) to tens of nanometers for planar micro-hotplates [14]. The nature of the deposition of platinum metal group catalysts (typically using Pt, Pd, Rh [15,16,17]) with ceramic catalyst carriers also changes compared to the use of direct metal particles without a carrier [18,19,20,21,22]. If, in the bead-type sensor, there is dripping to the already formed ball from the catalyst carrier by chemical reagents [12] and chemical reduction in Pt and Pd clusters due to the spiral’s micro-hotplate’s own heating [22], then with a decrease in particle size, clusters of catalytic material are deposited on the carrier particle powder before deposition on to the micro-hotplate in the form of a paste with organic binder. In the second case, the annealing function is primarily required to remove the organic binder from the gas-sensitive layer on the micro-hotplate after the initial deposition of the catalytic paste. This technological annealing, essential for eliminating organic residues, must be conducted at temperatures exceeding 700 °C (with 850 °C being the traditional benchmark for thick-film technology). However, these high temperatures pose a challenge for silicon MEMS technology, which is sensitive to mechanical stress in thin films.

Based on the aforementioned facts, the primary objective of this study is to develop a ceramic cantilever micro-hotplate that accommodates the deposition of a catalytic layer using the established bead-type design method. This approach ensures the retention of known and stable gas-sensitive properties during sensor operation. To enhance compatibility, the micro-hotplate’s power output, resistance specifications, and packaging form factor should ideally align with the existing series of sensors. This interchangeability is crucial, considering the finite service life of thermocatalytic sensors, which typically last between 2 and 3 years. Additionally, the packaging form factor plays a vital role in ensuring a high level of explosion protection [3,4,5,6], especially for applications in hazardous environments. The current research aims to create a ceramic micro-hotplate platform for thermocatalytic sensors that can be reliably fabricated at elevated temperatures (800–900 °C) and support the deposition of volumetric gas-sensitive materials.

## 2. Materials and Methods

**Figure 1:**
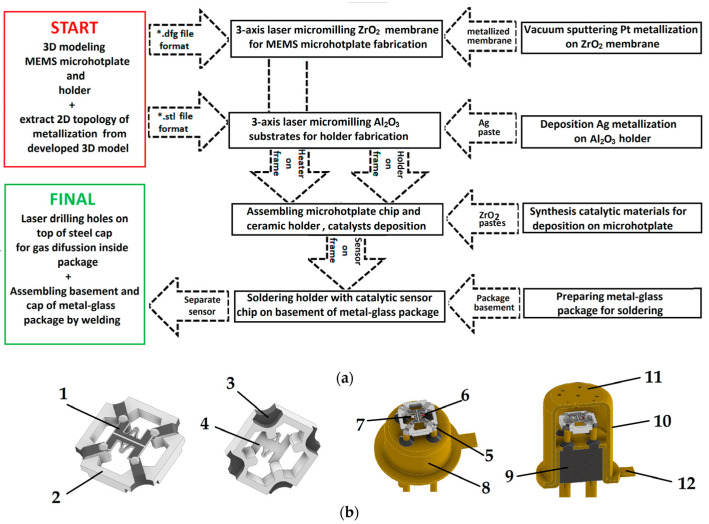
Technological steps used for the fabrication of thermocatalytic sensors: (**a**) Full flowchart for the fabrication of sensors; (**b**) 3D model of the thermocatalytic gas sensor in the TO-18 package used for fabrication.

To manufacture the ceramic components of the thermocatalytic gas sensor, we employed a digital technological workflow, as illustrated in Figure 1a. We developed a 3D model of the sensing device by using 3D modeling software COMPAS-3D Home (Russian Federation). As a result, the file was in STL format (as presented in Figure 1b). The design of thermocatalytic gas sensor in the TO-18 package consists of the following parts marked on Figure 1b: 1—Pt metallization; 2—Al_2_O_3_ ceramics; 3—Ag metallization; 4—ZrO_2_ membrane; 5—Sn/Pb solder; 6—active catalytic gas-sensitive bead; 7—reference catalytic gas-sensitive bead; 8—TO-18 package basement; 9—glass filling TO-18 package basement; 10—steel cap for TO-18 package; 11—holes in steel cap for gas diffusion to sensor element; and 12—key mark for side recognizing electrical contacts of active catalytic element.

**Figure 2:**
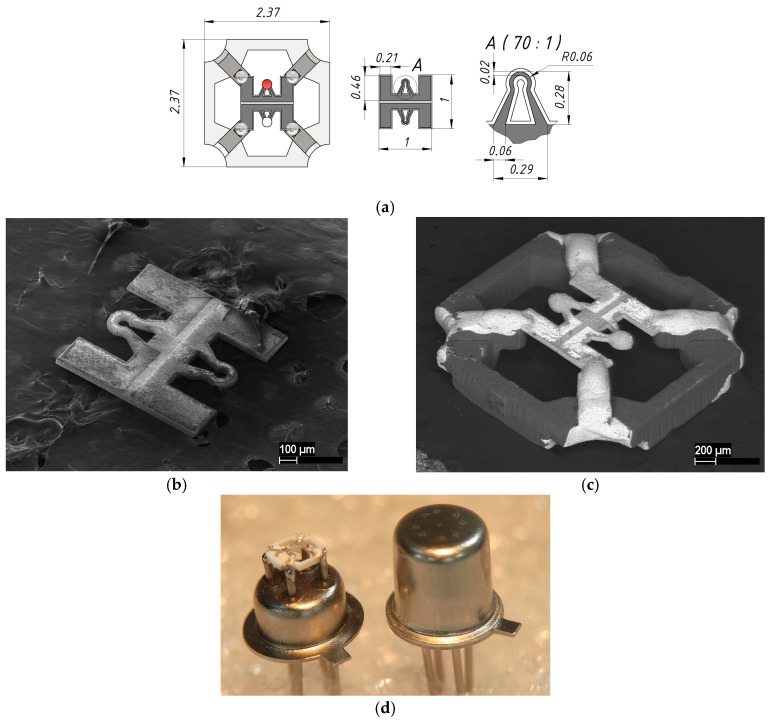
Appearance and dimensions of the thermocatalytic sensors: (**a**) sketch and topology dimensions of the cantilever type micro-hotplates; (**b**) SEM images of the ZrO_2_ membrane with a double micro-hotplate; (**c**) SEM images of the micro-hotplate mounted on the ceramic holder (dark colors show ceramic materials like ZrO_2_ and Al_2_O_3_ and light colors shows the metal layers); (**d**) optical photo of the soldered ceramic holder onto the TO-18 package basement (left) and the final sensor in a metal–glass package after steel cap welding (right).

The specialized 20 W fiber laser, featuring a tunable pulse duration of 50–200 ns and a wavelength of 1.064 µm, was employed to manufacture various components of the developed sensor, leveraging custom software for control [23]. This methodology enabled the integration of micromilling processes with digital comparisons of the fabricated devices, ensuring alignment with their geometrical specifications in the 3D model, as well as an assessment of the quality achieved post-fabrication. A thin ceramic ZrO_2_ membrane, measuring 1 × 1 mm and 30 µm in thickness, was fabricated from 3YSZ material (3 mol% Yttria Stabilized Zirconia). A cantilever design of a thin-film platinum heater, shaped like a horseshoe and exhibiting a resistance of 11–12 Ohms, was created using laser micromilling. Laser ablation of Pt metallization was carried out with power source—3%, scan speed—100 mm/s, and repetition rate—20 KHz, with pulse duration—100 ns and a laser spot diameter of 25 µm. During the platinum metallization laser ablation process, the ceramic membrane was also cut to ensure high accuracy in the topology of the micro-hotplate, enhancing the repeatability of each chip. The cutting mode was the same, but the number of laser beam passes was two orders of magnitude greater, providing high-accuracy topology for the micro-hotplate and repeatability for each chip. The topology of the micro-hotplate is shown in Figure 2. Platinum metallization with a thickness of 1 µm was deposited on a ZrO_2_ membrane by magnetron sputtering and annealed for several hours in an air atmosphere at 950 °C in order to stabilize the resistance of the micro-hotplate [24]. The resulting chip was mounted on a ceramic holder made of monolithic 96% alumina by adaptive laser micromilling [23], a photo of which is shown in Figure 2d. Laser micromilling of 0.5 mm thick alumina substrate was carried out with power source—90%, scan speed—300 mm/s, and repetition rate—20 KHz, with a pulse duration of 100 ns and a laser spot diameter of 25 µm. About sixty passes are enough to produce a complete full structure. The metallization of the ceramic holder is based on thick-film silver and has good soldering ability with standard solders (we used 60% Sn/40% Pb solid solder). The ceramic holder with a mounted chip based on the ZrO_2_ membrane was soldered onto a four-outlet metal–glass package holder TO-18 [25] at a height of 1.5 mm from the bottom of the package holder, thereby ensuring the location of the thermocatalytic elements in the middle of the inner volume of the metal cap. The metal cap of the package had a laser-perforated micro-hole for passing the gas mixture into the package to the micro-hotplates with active and reference catalytic elements. The holder and cap of the TO-18 metal–glass package were connected together by capacity welding, providing a sealed weld and an explosion-proof design for the sensor, thereby meeting the requirements for mass-produced products of this class.

**Figure 3:**
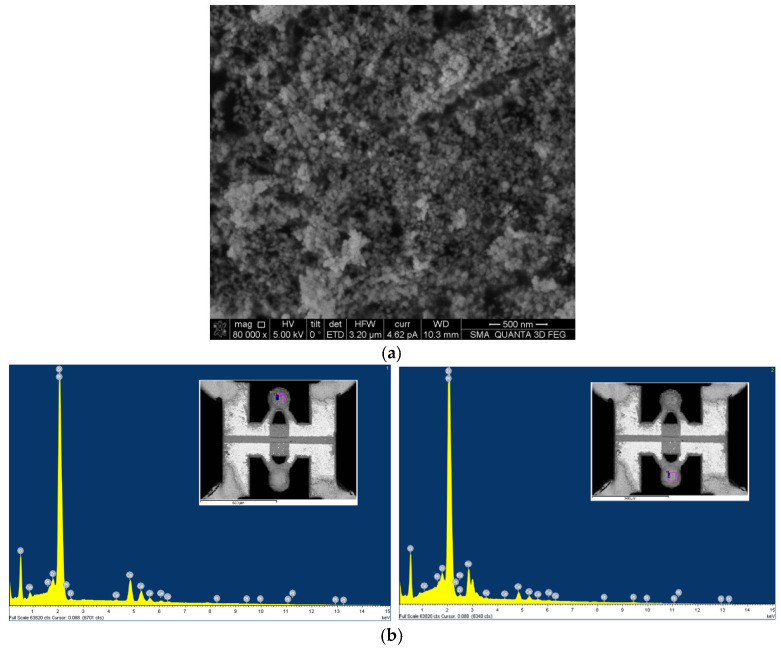
The CeO_2_/ZrO_2_ gas-sensitive material: (**a**) SEM image of initial ZrO_2_ carrier material; (**b**) element analysis of catalytic gas-sensitive material not covered (left) and covered by Pt and Pd catalysts (right).

A catalytic carrier of ZrO_2_ material with a particle size of 60–80 nm was chosen as the base (SEM image of carrier material presented in Figure 3a). The original ZrO_2_ material was impregnated by CeO_2_ and used for the deposition of Pt and Pd catalytic clusters. The advantage of adding CeO_2_ is that it can independently enhance the catalytic activity by the mechanisms described in the review in ref. [26]. Before chemical impregnation, a batch of CeO_2_/ZrO_2_ material was divided into two equal parts—one for synthesis of the active catalytic material and the other for the reference material, as a chemically inert element. This way, both elements had a similar surface area. In order to impregnate the catalyst support with the catalytic metal, salts of palladium chloride (PdCl_2_) and platinum hexachloro-acid (H_2_PtCl_6_) were used. During annealing at 500 °C, noble metal clusters formed in the catalyst support.

After the two components were combined with the terpineol-based binder, the resulting ink was prepared for drop coating onto the ceramic micro-hotplate. After mounting the holder with a ZrO_2_ membrane, we found that a catalytic gas-sensitive material based on nanodispersed ZrO_2_ was deposited onto the horseshoe-shaped micro-hotplate by drop coating. The final fabrication step involved firing the gas-sensitive material in air at 890 °C for 15 min (the same firing process was used for the Ag paste, which also incorporated a terpineol binder). Such harsh firing conditions were used due to the nano-size of the catalyst support, which creates unfavorable conditions for carbon extraction from deep layers of the granules.

**Figure 4:**
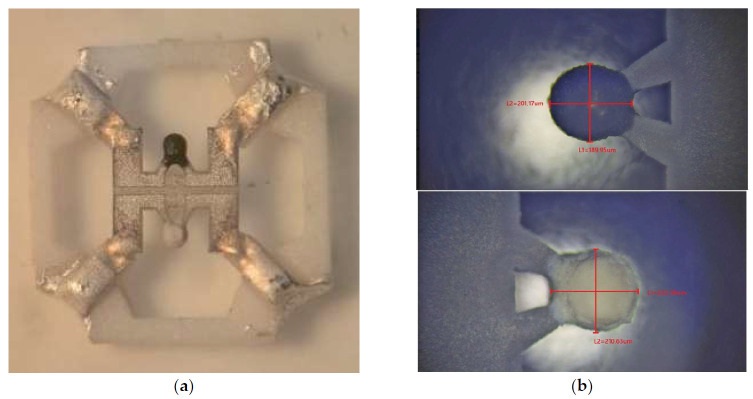
Images of the complete sensor element after deposition of gas-sensitive beads and before soldering on to TO-18 metal–glass package basement: (**a**) images from an optical microscope; (**b**) images from a digital measuring microscope, which can be used to judge the real dimensions of the fabricated catalytic beads elements (black bead in the top image containing Pt/Pd catalysts and white bead in the bottom image free from metal catalysts, with the natural color of ZrO_2_).

The SEM image and EDS element analysis of catalytic gas-sensitive materials (covered and not covered by Pt and Pd nano species) are shown in Figure 4b. Apart from the elemental compositions, a significant difference is found in the deposition of the two inks (the active and the reference material). The metal catalyst containing ink is better wetted by the organic binder, and therefore, we have a different mass of materials in the two types of inks with similar viscosity. To avoid using overly complex, yet effective, viscosity-related techniques, we used a digital optical microscope during gas-sensitive material deposition, trying to adjust the number of drops to achieve uniform ball diameters. The best result for the drop-by-drop uniformity of the beads (reference and active) is shown in Figure 4. Drying at 150 °C took place between droplet depositions for preventing cracking under the final sintering of formed beads. This was necessary, because the evaporation of organic binders is the main driver of cracking formation on the surface of beads.

**Figure 5:**
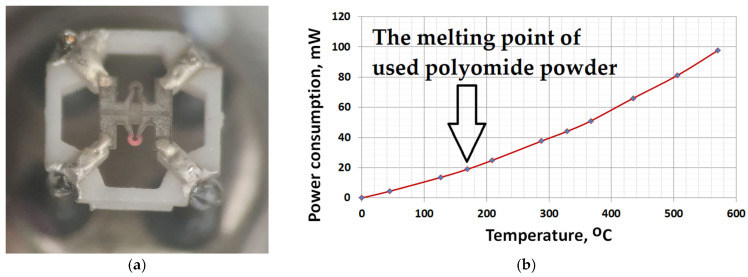
The thermocatalytic sensor during the heating process: (**a**) photo of heated micro-hotplate element (first element is emitting light, second one is cool for comparison); (**b**) dependence of power on operating temperature for one bead element.

One material was pure ZrO_2_ (a white bead)—a comparative element was formed from one bead—and the second material contained the same powder, onto which the Pt-Pd catalyst was deposited (a black bead). Figure 3 and Figure 4 show images from a scanning electron microscope and an optical measuring microscope, respectively, which can be used to accurately assess the true dimensions of the manufactured sensor. The size of the final beads is about 200 microns and has a less than ±10% scatter, because it is difficult to deposit the material without automatic equipment. The sensor elements were connected to the classical Wheatstone bridge electrical circuit used in thermocatalytic gas sensor tests [27]. The dependence of the output signal as a voltage difference in the two branches of the Wheatstone bridge electrical circuits vs. methane concentration was registered. The supply voltage for the sensor bridge was consistently set to 3.0 V. For the gas sensitivity test, methane gas was introduced to the sensor from a cylinder containing an air mixture with a methane concentration of 2.1% by volume. The measured response of the fabricated sensor at an operating temperature of 500 °C was 25 mV/%vol. CH_4_, with an energy consumption of 160 mW for both heating elements. Figure 5 illustrates the relationship between power consumption and temperature for a single sensor element coated with a catalytic layer. This dependence was validated using the micro-fusing technique with EOS PA2200 polyamide powder, characterized by a particle size of 50 microns [28]. For a more detailed description of the technique, please refer to [29].

## 3. Results and Discussion

Identical and reproducible platinum micro-hotplates were formed on a ZrO_2_ membrane, providing high mechanical strength of the system during long-term thermal cycling, since the coefficients of thermal expansion of the materials are almost identical—10.5 × 10^−6^ /K and 9.4 × 10^−6^/K, respectively (this value is one of the lowest among all metals, which makes platinum a very stable material when exposed to temperature fluctuations). The ZrO_2_ membrane cantilever design of the micro-hotplates was chosen for stress compensation to enhance mechanical stability and minimize bending relative to the original plane during unidirectional thermal expansion. This approach effectively reduces mechanical stress, thereby extending the sensor element’s service life.

To test the thermal coefficient of resistance for the fabricated micro-hotplate, a climate chamber KTH-74 [30] with a generated temperature range of −65 to +165 °C was used. The micro-hotplate soldered in a basement of a TO-18 package was placed inside the climate chamber, and the change in the micro-hotplate’s Pt metallization resistance was measured over the entire temperature range generated. The measured Pt metallization resistance changed linearly with a coefficient of 0.0218 1/°C. The resistance of the single micro-hotplate was approximately 11 Ohm at a temperature of 20 °C and varied slightly from sample to sample—resistance fluctuations for all manufactured elements were within 1 Ohm. The difference in resistance of the elements can be explained by the roughness of the manufacturing method—the laser ablation spot for Pt metallization was about 25 µm, with a wavelength of 1.064 µm for the laser source used. The de facto dimensions illustrated in the sketch in Figure 2a represent the maximum stable resolution achievable with the laser utilized in our micromachining technique. While the micro-hotplate resistance of the manufactured sensors matched that of widely used “pellistor” classical sensors in the studies [1,9,12], the thermal resistance coefficient was half that of a solid platinum wire (0.039 1/°C). This fact does not fully support the possibility of replacing the classic pellistor design of a catalytic thermal sensor (for the commercial market, interchangeability is important). An increase in the thermal resistance coefficient for the developed sensor design may be achieved by increasing the thickness of the initial sputtered platinum coating or by changing the topology (changing the ratio of hot and cold areas in the micro-hotplate).

The ceramic holder’s design, featuring thick-film metallization, enables high-temperature processing for the deposition of catalytic coatings prior to enclosing the entire assembly within a metal–glass TO-18 package. The critical feature of thick-film group operation in microelectronics is the need for annealing in an air atmosphere to remove the organic bond at 800–900 °C (the most critical temperature is 960 °C—the melting temperature of silver in the metallization on the ceramic holder). The holder with the mounted micro-hotplates successfully withstands such technological thermal cycling without the loss of electrical contacts at least several times, which makes it possible to deposit multiple catalyst layers onto the heater (with some variant layers against poisoning protection, as described in patent [31], or mechanical strengthening of beads, as in patent [32]). In our current work, the gas-sensitive layers were also deposited directly onto micro-hotplates mounted in the metal–glass package to demonstrate the possibility of annealing the gas-sensitive layer directly by using the power of the sensor element. Figure 5a shows a micro-heating plate heated to light radiation (over 600 °C). It is necessary to demonstrate the ability of the sensor element to operate at high temperatures to burn coke (carbon residues) periodically formed on the surface of the catalytic element due to insufficient oxidation of methane or other hydrocarbon gas due to a lack of oxygen [9]. In addition, the micro-hotplate area of the sensor element can be completely covered with a catalyst or a passive material, similar to bead-type sensors [3,4], which facilitates the deposition of the catalyst in various ways (ink or aerosol jet printing, etc.) and not only the way demonstrated in this work.

**Figure 6:**
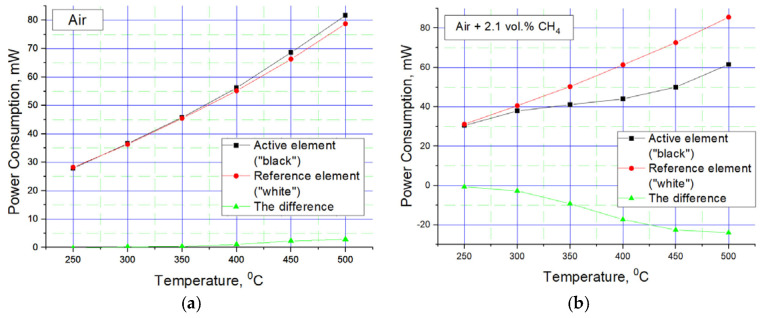
Dependence of the power consumption on the temperature of the active and comparative elements during operation: (**a**) in clean air; (**b**) in air mixture with methane, which gives 42% LEL (100% LEL is a concentration of 5% methane).

Despite the apparent primitiveness of its manufacture, this sensor design has an acceptable balance of elements—this can be seen in the graph of the power imbalance between the active and reference elements of the sensor. The results are measured with a 300 sccm flow rate (measurement by a mechanical rotameter), a chamber volume of Ø25×40 mm, an absence of humidity (direct flow from cylinder with pressed gas mixture), and an ambient temperature room temperature of 20 °C. Another advantage of the sensor design is shown in Figure 6b, where visible sensitivity to methane begins at 300 °C. This is due to the nano-dispersion of the catalytic carrier (confirmation given in the SEM image present in Figure 3a) and the additional presence of cerium oxide in the catalytic carrier. Also, the plot describing sensitivity to methane at 450–500 °C reaches relatively flat areas—this is also good, since there is an area in which sensitivity does not change much, in which it is possible to measure in a targeted manner with low error. The flat region on Figure 5b also has a positive influence on the slight technical drift of platinum, 1–2% per year. If there is a flat region in the response vs. temperature, then the small resistance drift limited by this region is unimportant. But if we have a response line with a large gradient in the region of gas temperature measurement, each low drift in resistance should raise the total error in measurement. Sometimes, an error in LEL concentration measurement could lead to a serious incident and human losses, especially in the mining or oil refectory industries.

## 4. Conclusions

The miniature ceramic ZrO_2_ micro-hotplate elements were designed and manufactured using laser micromachining techniques. This innovative method enables the rapid development of various gas sensor micro-hotplate designs—including membrane, bulk, and cantilever structures—not limited to thermocatalytic gas sensors. The technique has been successfully applied to different types of gas sensors, such as metal oxide [22], field-effect [33], and thermal conductivity [34] sensors, which utilize alternative principles of gas detection and sensing materials compared to thermocatalytic sensors.

The design of our thermocatalytic sensor platform (holder + micro-hotplate) closely resembles our previous work with the silicon MEMS [9]. However, this version is con-structed from ceramic materials, which simplifies the manufacturing process by eliminating the need for a clean room or intricate silicon MEMS fabrication techniques. This technological approach is particularly beneficial for newcomers to gas sensor development, while also serving experienced teams seeking advanced technology integration. Our approach also may facilitate the creation of more complex ceramic MEMS solutions for solid-state gas sensors, as demonstrated in works [35,36], which utilize ZrO_2_ micro-hotplate elements to combine different measurement principles.

For the final fabrication of the thermocatalytic sensor, a catalytic carrier composed of CeO_2_/ZrO_2_ nanomaterials was impregnated with Pt-Pd salts to create inks that facilitate the formation of active and reference suspensions during deposition onto micro-hotplate elements. We have demonstrated the feasibility of depositing gas-sensitive catalytic layers onto a unilaterally open cantilever structure of the micro-hotplates.

The newly developed design features micro-hotplates coated with catalytic and reference materials, creating typical microbeads similar to those found in traditional wire design thermocatalytic pellistors, with a diameter of approximately 200 microns. This innovative micro-hotplate design is more suitable for mass production, leveraging advanced microelectronic technologies, while maintaining the same volume of catalytic material as the classic wire pellistor design.

Given that the amount and quality of the catalytic material are consistent, previous studies suggest that the operational lifespan of the developed sensor could exceed one year at an operation temperature of at least 500 °C—a common verification interval for most gas analyzers utilizing thermocatalytic sensors [16]. Our future experiments will focus on assessing the sensors’ resistance to poisoning agents, such as siloxanes and sulfur, and evaluating their long-term stability. The high-temperature ceramic approach outlined in this work facilitates the development of bead-type multilayer gas sensing layer deposition technology, which is a crucial advancement for the evolution of advanced thermocatalytic sensors.

## Data Availability

The original contributions presented in this study are included in the article. Further inquiries can be directed to the corresponding author.
